# *Candida albicans* infection model in *Drosophila melanogaster* suggests a strain-specific virulent factor boosting a stormy innate immune response

**DOI:** 10.3389/fimmu.2024.1474516

**Published:** 2024-10-31

**Authors:** Mariona Cortacans, Marta Arch, Esther Fuentes, Pere-Joan Cardona

**Affiliations:** ^1^ Servei de Microbiologia, Laboratori Clínic de la Metropolitana Nord (LCMN), Hospital Universitari Germans Trias i Pujol (HUGTiP), Badalona, Spain; ^2^ Experimental Tuberculosis Unit (UTE), Institut de Recerca Germans Trias i Pujol (IGTP), Badalona, Spain; ^3^ Microbiology and Genetics Department, Universitat Autònoma de Barcelona, Bellaterra, Spain; ^4^ Centre de Medicina Comparativa i Bioimatge de Catalunya (CMCiB), Badalona, Spain; ^5^ Centro de Investigación Biomédica en Red de Enfermedades Respiratorias (CIBERES), Madrid, Spain

**Keywords:** priming, innate immunity, *Drosophila melanogaster*, infection, sexual dimorphism, pathogen load, β-glucan, environmental mycobacteria

## Abstract

**Intorduction:**

Pathogens drive the evolution of host defence strategies, with both innate and adaptive immune systems playing key roles. Priming enhances the innate immune system’s readiness by functionally reprogramming immune cells after initial exposure to stimuli, like β-glucans. In this sense, *Drosophila melanogaster* is a valuable model to evaluate the role of innate immunity to control infections.

**Objectives:**

In this study we aimed to set light on the immune priming effect of oral treatment with heat-killed *M. manresensis* and two different heat-killed *C. albicans* isolates upon systemic infection by *C. albicans* in the *D. melanogaster* model.

**Methods:**

A clinical and a control ATCC 90028 *Candida albicans* strain were used. Flies were primed through oral administration of heat-killed *C. albicans* (hkCa), both clinical and control, and hk-*Mycolicibacterium manresensis*. After priming, flies were systemically infected with both *C. albicans* isolates. Host survival, pathogen load, and immune response in response to treatment and infection were evaluated.

**Results:**

Both treatments showed a significant capacity to enhance the expression of antimicrobial peptides, in particular Diptericin, and Drosomycin in males. This response had a marked sexual dimorphism due to the difference in Upd3, Nox, and Duox expression. Surprisingly, even when priming was able to avoid the growth of both *C. albicans* strains, survival was not improved in the case of the clinical isolate, causing an unexpected mortality rate in hours, regardless of the host’s sex. Gene expression analysis 24 hours post-infection showed an exacerbated increase in Diptericin, Drosomycin and Upd3 expression upon infection with the clinical strain.

**Conclusion:**

Data herein suggests the presence of a strain-specific component in *C. albicans* as the booster of a “stormy” innate immune response, which must be further investigated, and position *D. melanogaster* as a useful model for evaluating virulent factors related to the modulation of the innate immunity.

## Graphical Abstract

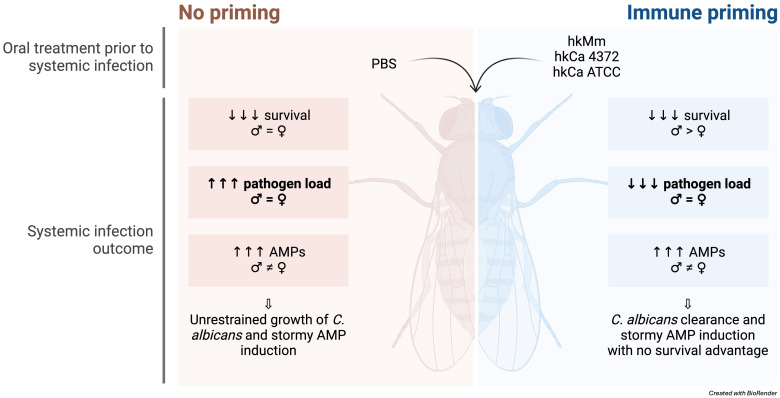

## Highlights

- Oral priming with heat-killed *Mycolicibacterium manresensis* and *Candida albicans* induces antimicrobial peptide production and pathogen load reduction.
- Systemic infection with the clinical isolate *C. albicans* 4372 results in an extensive AMP production and rapid host mortality.
- Male and female flies present distinct patterns of AMP induction in response to immune priming and infection.
- Primed male flies present higher survival rates than females after infection with *C. albicans* 4372.

## 1 Introduction

Pathogens are widespread and have driven the evolution of host defense strategies like resistance and tolerance (1, 2). When a pathogen is able to bypass host defenses, the host can combat the infection by clearing the pathogen or by targeting its replication rate, although this response can be resource-intensive and may cause self-damage (1). Historically, the vertebrate immune system has been divided into innate and adaptive arms (3). Following this duality, it was assumed that the innate arm was focused on a rapid and non-specific response, while immunological memory was the hallmark of the adaptive response (3–5). However, the growing evidence indicating that innate immune cells can display adaptive traits is challenging this dogma (3, 5).

Initial exposure to a pathogen can lead to immunological memory, providing individuals with increased protection upon a secondary exposure to the pathogen (i.e., challenge) (1). Greater protection against reinfection has been reported not only in mammals (3–5), but also in plants and invertebrates, which do not possess adaptive immune responses (3, 6–9). Among the different adaptive programs that can be induced in innate immune cells, the modulation of innate immunity can be either positive (i.e., immunopotentiation) or negative (i.e., immunosuppression) (10, 11). Specifically, priming is a form of immunopotentiation that leads to the functional reprogramming of innate immune cells after initial exposure to an immunomodulator, and active gene transcription does not return to its basal levels before the second challenge (4, 9, 12). Regarding the priming agents, polysaccharides from plants and microorganisms, including β-glucans, are particularly significant (10, 13–15).

β-glucans are heterogeneous polysaccharides found in yeast and fungal cell walls, not produced by mammalian cells (15). Since the 1940s, β-glucans have been investigated as immune modulators, and today, oral supplementation with β-glucans is widely used for tissue regeneration, microbiome modulation, enhancing inflammatory responses, and trained immunity (11, 15). Conversely, *Mycolicibacterium manresensis* is a fast-growing environmental mycobacterium that belongs to the *M. fortuitum* complex and has shown low toxicity in mice and humans (16). Recent studies present evidence of heat-killed *M. manresensis* (hkMm)-dependent trained immunity induction by THP-1 and primary monocyte cells in response to lipopolysaccharide (LPS), *Staphylococcus aureus, Mycobacterium tuberculosis*, and SARS-CoV-2 (17).

Several animal and plant models have been developed to study these interactions. Specifically, *Drosophila melanogaster* has provided huge insights on innate immune response mechanisms, since insects rely merely on this type of response (4, 18, 19). This, together with the highly-conserved immunological features of the fruit fly in mammals, including a high number of disease-causing gene homologs, is what makes *D. melanogaster* a powerful model to study innate immune responses and gene functionality (2, 4). To combat infection, *D. melanogaster* relies on cellular and humoral innate immune responses, involving phagocytosis and encapsulation by immune cells (predominantly haemocytes) and antimicrobial peptide (AMP) and reactive oxygen species (ROS) production (18–21). *D. melanogaster* has been extensively used to study trained immunity against an infective challenge, as reviewed by Arch et al. (1, 4). Nevertheless, the role of *M. manresensis* as an immunomodulator against infections has yet to be defined in this model.

In this study, we aimed to set light on the immune priming effect of oral treatment with heat-killed *M. manresensis* and two different heat-killed *C. albicans* isolates upon systemic infection by *C. albicans* in the *D. melanogaster* model. To do so, host survival, pathogen load, and gene expression in response to treatment and infection were evaluated.

## 2 Materials and methods

### 2.1 Fly maintenance

Nutri-Fly^®^ Bloomington Formulation (Genesee Scientific, USA) media was used to feed the flies, prepared following manufacturer’s instructions at 24 ± 1°C and 70% humidity. After being autoclaved, 2 mL of propionic acid were added to prevent yeast contamination. *D. melanogaster* Oregon-R-C were raised and maintained in 250 mL polypropylene bottles (57 x 103 mm) with 50 mL of media, while polystyrene tubes (28.5 x 99 mm) with approximately 10 mL of media were used for experimental procedures.

Culture tubes were changed every 3-4 days during experiments, and empty tubes with media were stored at room temperature for up to one month. Flies were kept at 25°C with constant light:dark cycles of 12 hours each and 70% humidity, as *Drosophila* immunity is influenced by circadian rhythms. To avoid ageing bias during experiments, it was essential that all flies were the same age. To ensure this, 30 males and 30 females were placed into new bottles for 24 hours, allowing the females to lay eggs, after which adults were removed and the empty bottles were kept at 25°C. New flies emerged after 10 days, guaranteeing that all of them were born on the same day.

### 2.2 Strains

Two *C. albicans* strains were used in this study: *C. albicans* ATCC 90028, a reference strain, and *C. albicans* 4372, a clinical isolate obtained from a pleural fluid sample. Both present intrinsic resistance to ampicillin, and they were kindly provided by the Microbiology Department of Hospital Universitari Germans Trias i Pujol (HUGTiP). In the case of *M. manresensis*, it has intrinsic resistance to doxycycline.

### 2.3 Experimental design

**Figure 1:**
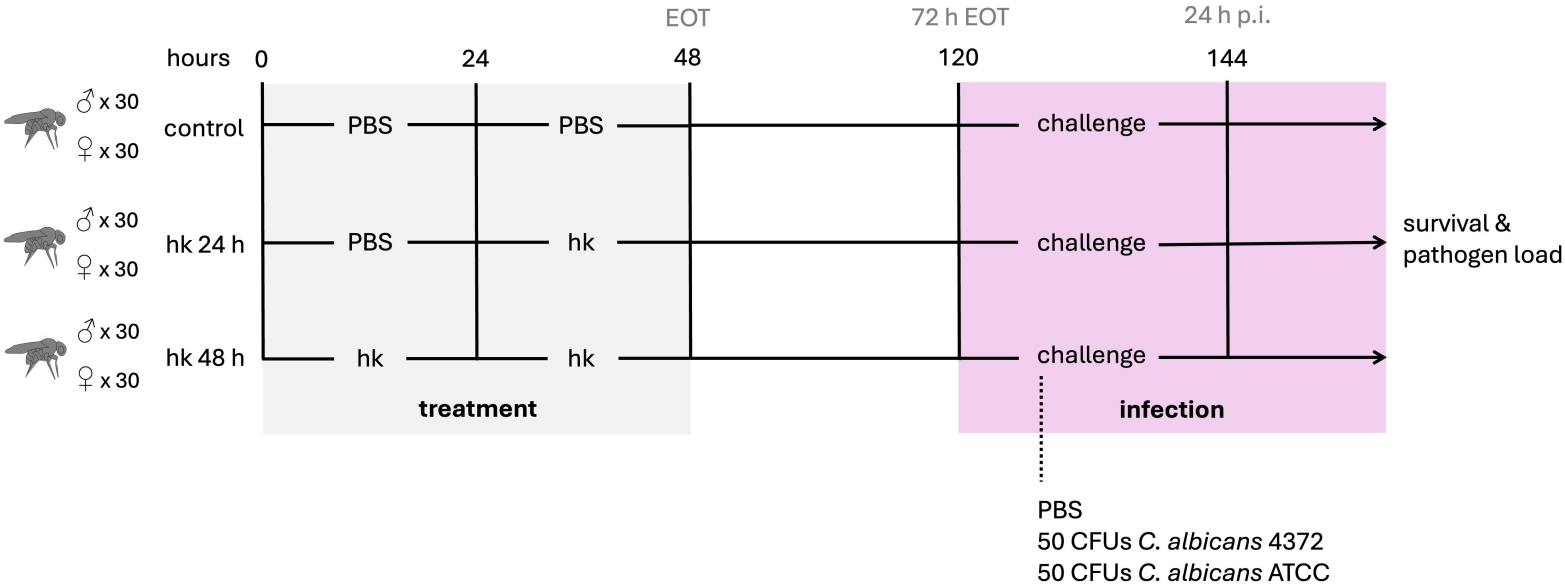
Experimental design. Male and female flies were orally exposed to heat-killed *M. manresensis*, *C. albicans* ATCC or *C. albicans* 4372 for 24 or 48 hours. Oral exposure to sterile PBS was used for untreated controls. After treatment, flies were fed with normal media for 72 h before infection with both *C. albicans* strains or sterile PBS. Survival and pathogen loads were followed after infection and gene expression was evaluated pre- and post-infection.

To study the effect that oral administration of heat-killed *M. manresensis* (hkMm) and *C. albicans* (hkCa) could have on infection progression with the aforementioned yeast isolates, male and female flies in equal proportions were orally exposed to hkMm and hkCa, placed on top of the fly medium, for 24 and 48 hours. For the 48-hour groups, new hkMm and hkCa was added at 24 h. Both treatment regimens were included to assess whether there was a dose-dependent effect on the priming response, and also because female flies did not respond to the treatment during the first 24 hours (data not shown). For the untreated groups, fly medium was supplemented with phosphate-buffered saline (PBS). After treatment, flies were fed with normal medium for 72 h before injection with 13.8 nL of either PBS or *C. albicans* at a final concentration of 50 CFUs/fly. This was the time point that we identified corresponded to the peak of the priming response. The experimental design for this study is outlined in **Figure 1**.

### 2.4 Heat-kill preparation

One aliquot of each isolate was defrosted and centrifuged 5 min at 5,000 g. Supernatants were discarded to remove residual glycerol, and pellets were resuspended with 100 μL of sterile PBS. The samples were then inactivated at 80°C during 20 min, after which all microorganisms were dead. 75 μL containing 10^5^ CFUs of each heat-killed sample were then inoculated in fly maintenance tubes, on top of the fly media. Tubes were left to dry overnight before introducing the flies.

### 2.5 Assessment of hkMm intake

**Figure 2:**
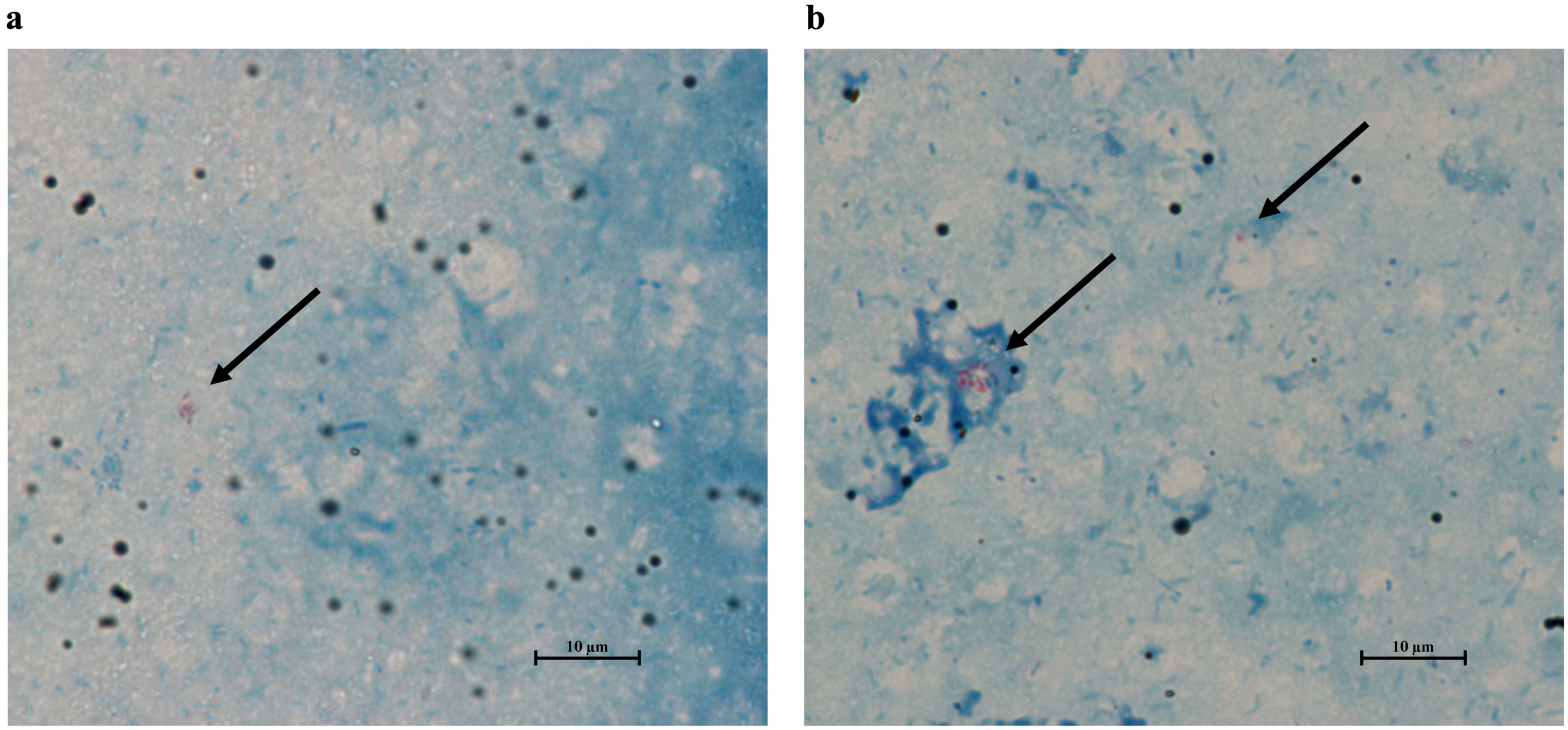
Ziehl-Neelsen stain of the gut of flies treated with heat-killed *M. manresesis*. Both male and female flies contain acid-fast bacteria in their guts 24- **(A)** and 48-hours **(B)** after the start of the treatment. Arrows point to the *M. manresensis* clusters in the samples, and their presence confirms oral treatment effectiveness. Pictures taken with the NIS-Elements D Software (Nikon Instruments Inc, version 5.11.03).

To evaluate whether flies were ingesting the hkMm treatment, 5 male and 5 female flies were selected at 24- and 48-hours after the start of the oral treatment. After being anaesthetized, the heads and wings of the selected flies were removed using a razor blade. Flies were then transferred into a glass slide and coated with cold PBS to keep the tissues hydrated. Then, for each fly, one pair of tweezers was used to grasp the thorax, while a second pair was used to gently pull the posterior end of the abdomen, until the entire gut was removed from the abdominal cavity. This process was repeated five times, and the resulting guts were homogenized into 50 μL of PBS. Three 5 μL drops of each preparation were placed into glass slides and further stained using the Ziehl-Neelsen kit (BD BBL™ TB Stain Kit ZN). Slides were observed under the 100X objective of an optical microscope, and treatment was considered successful after confirming the presence of acid-fast bacilli in the preparations (**Figure 2**).

### 2.6 Systemic infection

Systemic infections were performed on 3 to 5-days-old flies. Stock aliquots were defrosted, centrifuged 5 minutes at 5,000 g and resuspended in PBS before diluting them to the desired infective dose. Flies were anaesthetized using CO_2_ and injected in the ventrolateral surface of the anterior abdomen with 13.8 nL of the infection mix, using Nanoject II^®^ (Drummond). All infection solutions consisted in a 50 μL volume mix of either PBS or the designated microorganism and Brilliant Blue. Survival of the control groups was monitored up to 2 days post-infection for wounding control, and flies that died within these days were eliminated from the experiments.

### 2.7 Pathogen load analysis

Dead flies were collected from the maintenance tubes and transferred to individual eppendorf tubes. Each fly was then washed with 70% ethanol, rinsed with PBS, and homogenized into 200 μL of sterile PBS. Serial dilutions were then performed and 5 μL of each dilution were spotted in Luria-Bertani (LB) plates supplemented with ampicillin to select for *C. albicans*. Plates were incubated 24 h at 37°C, and viable bacteria were quantified by counting colony forming units (CFUs) after incubation time. It is worth noting that this technique carries an intrinsic detection limit of 40 CFUs due to the low sample volume cultured.

### 2.8 Gene expression analysis

#### 2.8.1 Sample preparation

Flies from each experimental condition were harvested in cold ethanol to permeabilize them and washed once with cold PBS afterwards. 150 μL of RNA Later (Invitrogen) were then added to each tube and left overnight at 4°C. Finally, they were transferred to -80°C until processed. To test reproducibility, each essay was performed with technical triplicates for each biological sample and three replicates for each experimental condition.

#### 2.8.2 RNA extraction, purification, and quantification

Total RNA was extracted using the MasterPure™ Complete DNA and RNA Purification Kit (Lucigen), working on ice throughout the whole procedure. To further calculate the exact amount of RNA needed for the retro-transcription reaction, all samples were quantified using NanoDrop^®^ ND 1000 Spectrophotometer (Nanodrop Technologies Inc., Wilmington, DE, USA). Inclusion criteria for posterior amplification were a A_260_/A_280_ ratio between 1.8 and 2 and a A_260_/A_230_ ratio between 2 and 2.2.

#### 2.8.3 cDNA synthesis and real-time PCR assay

The extracted RNA was converted to cDNA with the PrimeScript RT Master Mix (Takara). Real-Time (RT) quantitative PCR (qPCR) was carried out in in the LightCycler^®^ 480 II (Roche, F. Hoffmann-La Roche, Ltd), using 10 μL reaction solutions containing 5 μL of KAPA SYBR^®^ FAST Mix (Sigma), 2 μL of cDNA (diluted 1:3), 0.1 μL of each specific primer and 2.8 μL of water. PCR conditions consisted of 95°C for 5 min followed by 40 cycles of 95°C for 10 sec and 60°C for 20 sec. For *upd3* and *nox*, the amplification cycles were carried out at 62°C and 57°C -respectively- instead of 60°C. Finally, to detect amplification of *duox*, elongation cycles of 20 sec were used. The relative transcript levels of target genes were calculated using the 2^-ΔΔCT^ method (22), with *rpl32* as the reference gene for normalization of target gene expression abundance. The oligonucleotide sequences used for Real-Time PCR (23, 24) are displayed in **Table 1**.

**Table 1:** Oligonucleotide sequences used for Real-Time PCR.

Gene	Primer forward (5’-3’)	Primer reverse (5’-3’)	Ref.
*rpl32*	ACAGGCCCAAGATC GTGAAG	TCGACAATCTCCTT GCGCTT	(19)
*diptericin*	GGCTTATCCGATGC CCGACG	TCTGTAGGTGTAGGT GCTTCC	(19)
*drosomycin*	CCAAGCTCCGTGAG AACCTT	CAGGTCTCGTTGTC CCAGAC	(19)
*upd3*	GCAAGAAACGCC AAAGGA	CTTGTCCGCATT GGTGGT	(19)
*nox*	GGCTATCTCCTGCA AGATCG	CCAACTCAATCAGG CGGTAT	(20)
*duox*	TTTGGATAGGTGTG CTGCGT	CGCTTTCGTTGAGG GGGATA	This study

### 2.9 Statistical analysis

Data were graphed and analyzed using GraphPad Prism [version 9.5.1 (733)]. The ROUT test was applied in all groups to identify and subsequently reject one or multiple outliers. CFU counts and gene expression data were checked for normality and lognormality with Shapiro-Wilk normality test. Multiple comparisons for data with Gaussian distributions were conducted using one-way ANOVA, while Mann-Whitney tests were applied for data with non-Gaussian distributions. On the other hand, for two-group comparisons, unpaired t-tests were conducted, while Kruskal-Wallis tests were used to analyze non-normally distributed groups. For the principal component analysis (PCA), RStudio (version 2023.12.1 + 402) was used to analyze the multivariate relationships within the datasets. An initial outlier test was applied to identify and subsequently reject outliers, and the ‘mt’ and ‘ggplot2’ packages was then used to analyze and visualize the resulting data, respectively. GraphPad Prism was used to graph variable contributions and to perform statistical analysis of the principal component coordinates. The p-values ≤ 0.05 (*), ≤ 0.01 (**), ≤ 0.001 (***), and ≤ 0.0001 (****) were considered significant (same for #).

## 3 Results

### 3.1 *Mycobacterium manresensis* is present in the gut of flies treated with hkMm for 24 and 48 hours

The dissection protocol confirmed the presence of *M. manresensis* in the gut of treated flies (**Figure 2**), and hereby demonstrates the success of the both 24-hour and 48-hour oral treatments with hkMm. However, this same methodology could not be applied to flies treated with hkCa, due to the fact that the intestinal microbiota of *D. melanogaster* is already rich in yeast species.

### 3.2 Oral administration of hkMm and hkCa induces Nox-dependent production of ROS

**Figure 3:**
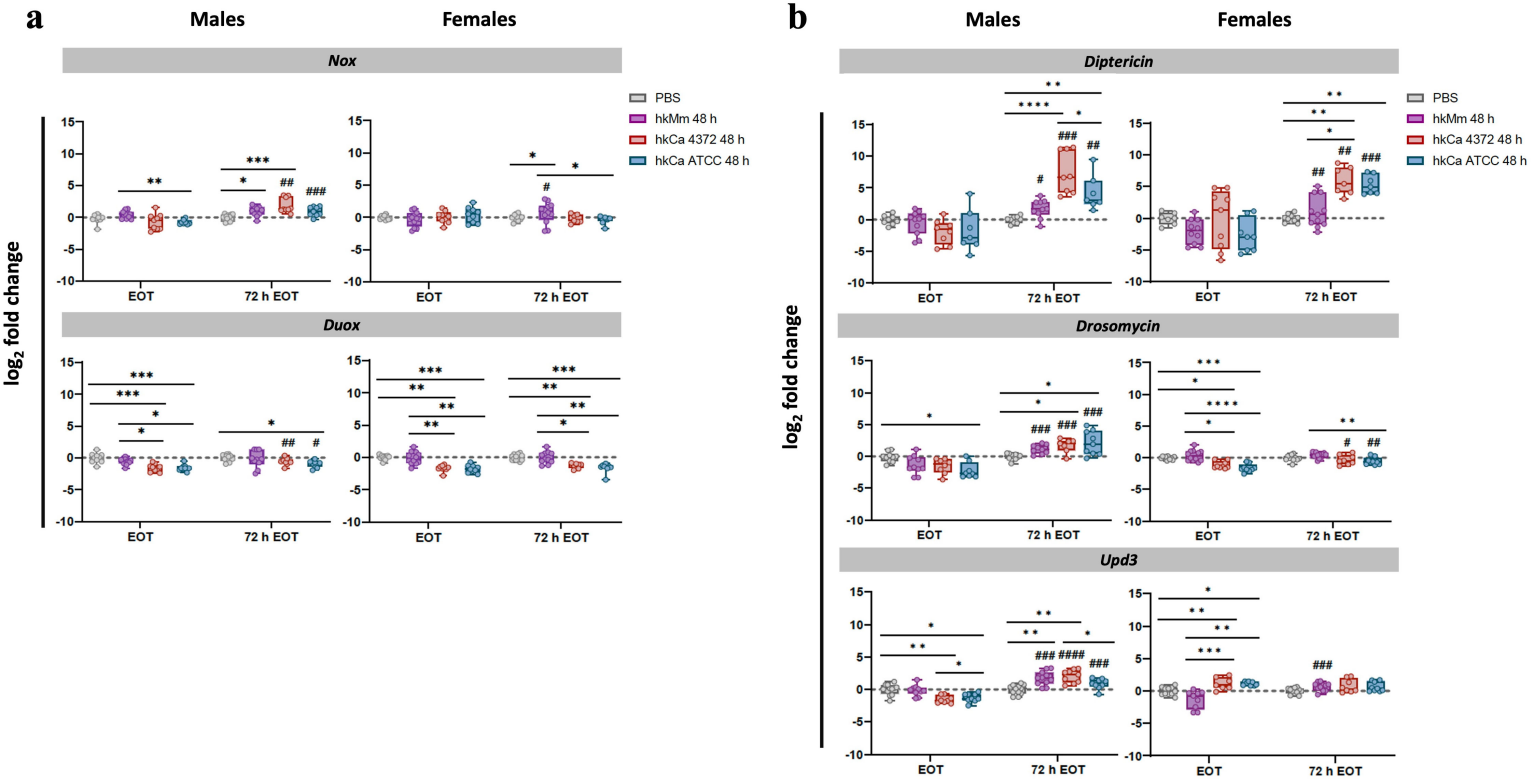
Gene expression analysis in response to the 48-hour treatment regimens. **(A)** Nox and Duox. **(B)** *Diptericin*, *drosomycin*, and *upd3*. Real-Time qPCR results were normalized with the *rpl32* gene and presented as the log_2_ fold change between the PBS-injected and pathogen-infected groups. Each dot represents the relative expression of the corresponding gene from a pool of three flies. The dashed line in each graph represents the controls’ relative expression with a fold change of 1. ‘*’ indicate differences between treatments within each time-point, while ‘#’ represent differences between each 72 h EOT sample and their respective EOT time-point. Data was analyzed for normality and significant differences were represented as follows: *p ≤ 0.05, **p ≤ 0.01, ***p ≤ 0.001, and ****p ≤ 0.0001 (same for #). For ‘*’ analyses, one-way ANOVA was used for normally distributed data, while Kruskal-Wallis test was used for non-parametric distributions; for ‘#’ analyses, unpaired t-test was used for normally distributed data, while Mann-Whitney test was used for non-parametric distributions.

We evaluated the effect of the oral treatment in the expression levels of two different NADPH oxidase enzymes (i.e., Nox and Duox), which are responsible for the production of reactive oxygen species (ROS) in *D. melanogaster*. Results for the 48-hour regimens are shown in **Figure 3**, while results for the 24-hour regimens in **Supplementary Figure S1**. None of the tested treatments induced significative NADPH oxidase enzyme production at the end of their respective regimens. However, both the 24-hour and the 48-hour hkMm administrations lead to an increase in *nox* expression 72 hours after the end of the treatments (72 h EOT) in males and females, although only male flies treated with hkCa displayed increased levels of *nox* induction in the 72 h EOT time-point. Regarding *duox*, no significative increase in expression was detected in flies treated with hkMm, while a significative reduction was detected in both hkCa treatment groups.

### 3.3 Male and female flies present different patterns of gene expression in response to treatment

To assess whether response to oral treatment with hkMm and hkCa showed sexual dimorphism in flies, a principal component analysis (PCA) was performed with the relative expression levels of treated flies. Results are shown in **Supplementary Figure S2**. Differences related to sex were mainly driven by the differential *nox* and *drosomycin* expression in the 72 h EOT time-point.

In males, an increase in *diptericin, drosomycin*, and *upd3* expression was observed at 72 h EOT in all treatment regimens in a dose-dependent manner. Results for the 48-hour regimens are shown in **Figure 3**, while results for the 24-hour regimens in **Supplementary Figure S1**. Even when the treatment was given orally and thus only the Imd pathway should be stimulated, data show both Imd (*diptericin*) and Toll (*drosomycin*) stimulation, together with Upd3, while females showed only stimulation of *diptericin* 72h EOT time-point.

**Figure 4:**
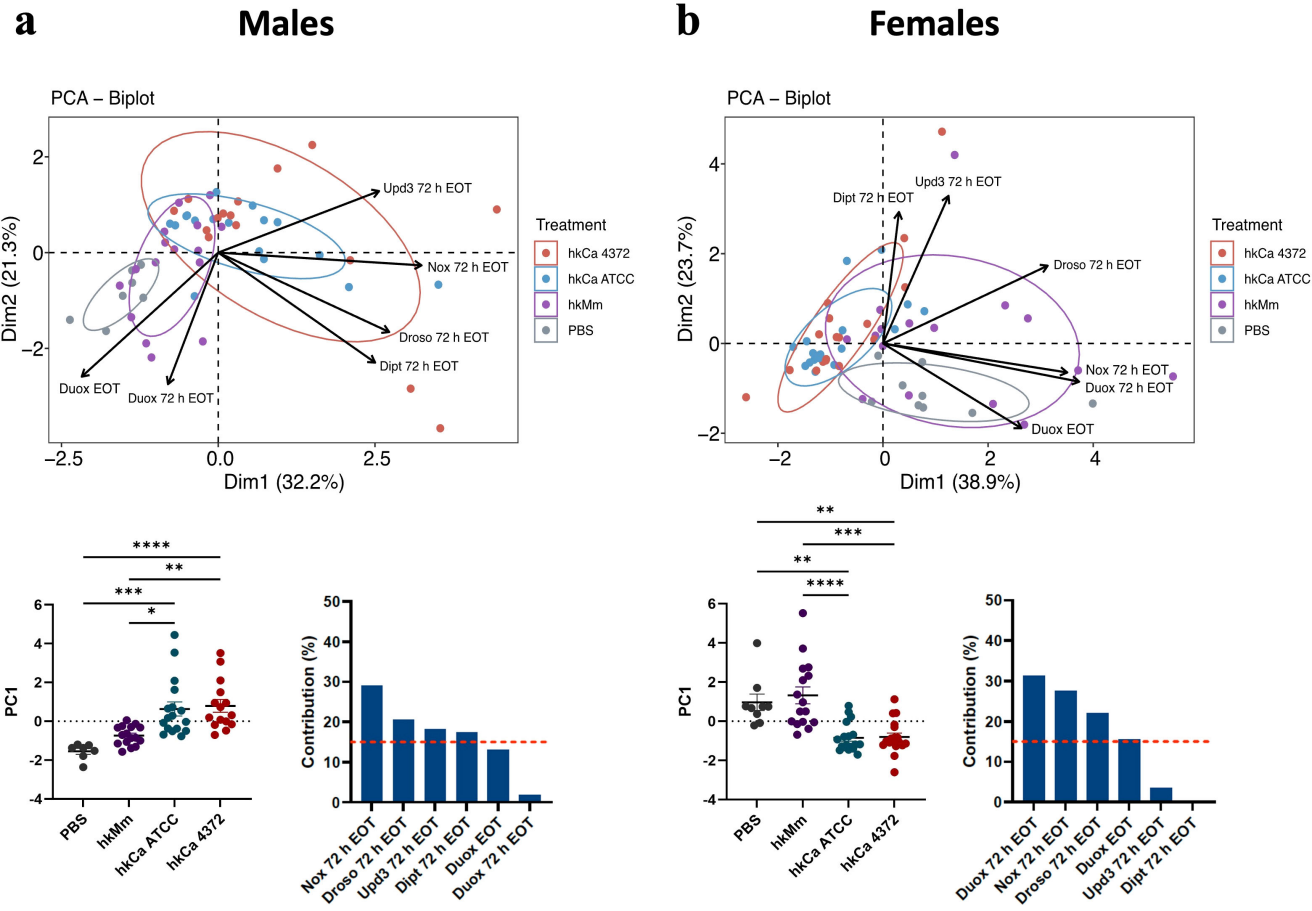
Disparities in treatment-induced antimicrobial peptide production according to initial stimuli. Principal component analysis (PCA) based on expression of selected genes in flies treated with hkMm and hkCa. (Top) Two separate clusters are generated, one containing hkMm and PBS and the other grouping both hkCa regimens in both males **(A)** and females **(B)**. (Bottom) PC1 scores (left) and variable contributions of Dim1 (right). In the PC1 scores, each circle represents an individual fly and lines depict the means in each group. Statistically significant differences are represented as follows: *p ≤ 0.05, **p ≤ 0.01, ***p ≤ 0.001, and ****p ≤ 0.0001 (one-way ANOVA for normally distributed data; Kruskal-Wallis test for non-parametric distributions).

Interestingly, PCA analysis (**Figure 4**) revealed that *nox* expression in males was the key differentiating component for the generation of two separate clusters: one containing both hkCa treatments, and the other grouping hkMm and PBS (i.e., absence of treatment) (**Figure 4A**). The same two clusters described in males could be distinguished in females, although in this case *duox* induction also played an important part in differentiating said clusters (**Figure 4B**). In this case, since no significant differences were observed between the 24- and 48-hour treatment regimens of each treatment group (**Supplementary Figure S3**), results were pooled to simplify data visualization.

### 3.4 hkMm and hkCa administration prompt *C. albicans* infection clearance

**Figure 5:**
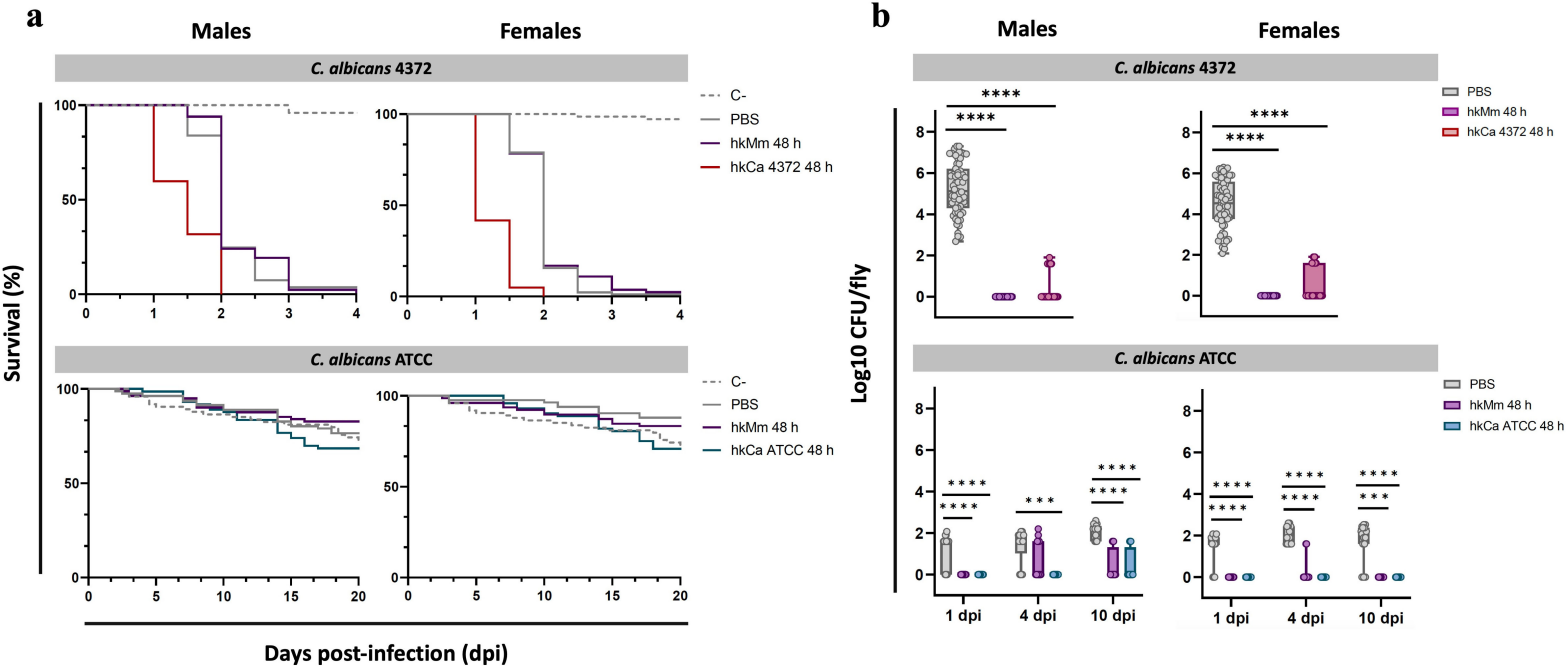
Rapid fly death due to infection with *C. albicans* 4372 and infection clearance in response to treatment. **(A)** Survival curves of flies infected with *C. albicans* 4372 and *C. albicans* ATCC (48-hour treatment regimens). *C. albicans* 4372 (Top) presented a highly virulent profile in both males (left) and females (right), while *C. albicans* ATCC (Bottom) displayed an avirulent phenotype. **(B)** Pathogen load of flies infected with *C. albicans* 4372 and *C. albicans* ATCC (48-hour treatment regimens). Pathogen load is expressed as Log_10_ CFUs/fly and each circle represents an individual fly. The limit of detection for bacterial colonies in each sample is set at 40 CFUs. Absence of CFUs was computed as a 1 instead of 0 to improve data visualization. (Top) Within the *C. albicans* 4372 infection group, pathogen load is represented as the Log_10_ CFUs counts at the moment of fly death. (Bottom) Within the *C. albicans* ATCC infection group, multiple time-points were set to determine pathogen load of live flies. Statistically significant differences are represented as follows: ***p ≤ 0.001, and ****p ≤ 0.0001 (one-way ANOVA for normally distributed data; Kruskal-Wallis test for non-parametric distributions).

To determine whether oral administration of hkCa and hkMm confers specific and non-specific protection to *D. melanogaster* against subsequent *C. albicans* infection -respectively-, flies were systemically infected with 50 CFUs of *C. albicans* 72 h after the end of the oral treatments. Results for the 48-hour regimens are shown in **Figure 5**, while results for the 24-hour regimens are displayed in **Supplementary Figure S4**.

Infection with *C. albicans* ATCC and shame presented similar survival curves, while flies infected with *C. albicans* 4372 displayed with a far more aggressive profile, with an average lifespan of 1.79 days post-infection (**Figure 5A**). Systemically infected flies with *C. albicans* 4372 after the end of the tested treatments did not display significant differences in survival compared to untreated flies. However, a drastic decrease in pathogen load was detected in males and females of all tested treatment regimens, with a greater impact in the hkMm 48 h group (**Figure 5B**). Thus no relation between pathogen load and survival could be demonstrated.

Similarly, systemically infected flies with *C. albicans* ATCC presented no significant differences in survival between treatment groups. Regarding CFU counts of dead flies, no differences were observed between groups (data not shown). Thus, multiple time-points were established to monitor the pathogen load of live flies. In this regard, significant differences between treated and un-treated groups were observed at 1-, 4-, and 10-days post-infection in both males and females, with no differences between 24- and 48-hour treatment regimens (data not shown). At 1-day post-injection, infection was only established in the untreated (i.e., PBS) group, and CFUs in these flies remained fairly stable during the following time-points. Regarding treated flies, infection progressed at 4-days post-infection only in the hkMm group in males, while remaining undetectable in hkCa ATCC. Finally, at 10-days post-infection, a few CFUs were detected in treated male flies, while females continued to exhibit absence of *C. albicans* infection.

In light of the high mortality detected in the *C. albicans* 4372 group, we injected 50 CFUs of hkCa 4372 into the flies’ abdomens in a preliminary evaluation with only 30 males and 30 females to ascertain if the observed rapid host death was caused by either the inflammatory response of the flies or a structural component of the pathogen or an exoproduct secreted by *C. albicans*. Results are displayed in **Supplementary Figure S5**. Survival was monitored up to 10 days post-infection, and fly lifespan remained comparable to the control group, indicating that *C. albicans* 4372 needs to be viable to cause the previously observed fly death.

### 3.5 Infection with *C. albicans* 4372 leads to sexually-dimorphic and stormy AMP induction

**Figure 6:**
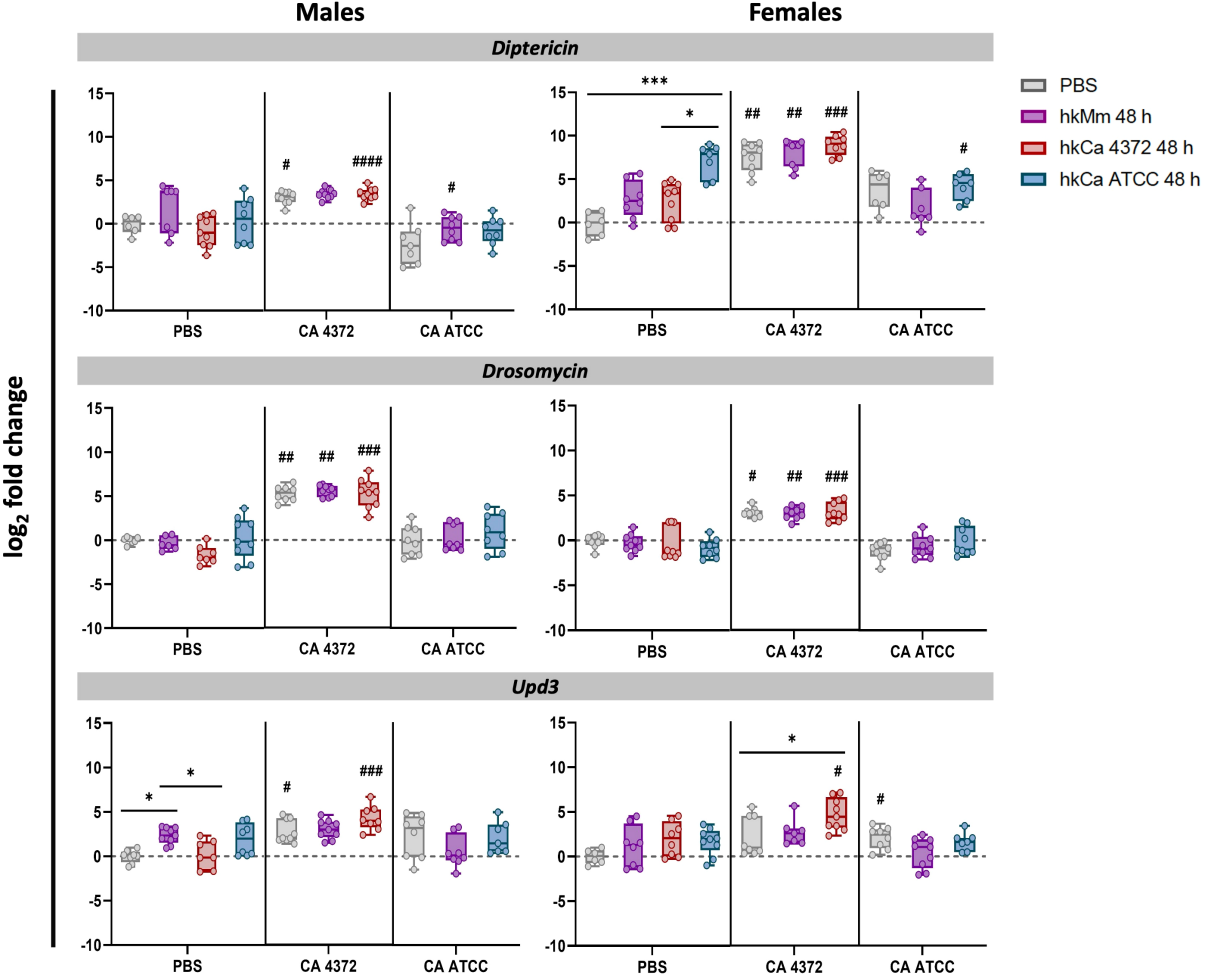
*Diptericin*, *drosomycin*, and *upd3* expression 24 h post-infection (48-hour treatment regimens). *C. albicans* 4372 prompted high *diptericin*, *drosomycin*, and *upd3* expression in both males and females, in contrast to *C. albicans* ATCC. Expression levels were calculated using the 2^-ΔΔCT^ method with *rpl32* gene for normalization. ‘*’ indicate differences between treatments within each time-point, while ‘#’ represent differences with their relative uninfected control after the treatment to discern expression variations due to infection. Each dot represents the relative expression of the corresponding gene from a pool of three flies. The dashed line in each graph represents the controls’ relative expression with a fold change of 1. Data was analyzed for normality and statistically significant differences are represented as follows: *p ≤ 0.05, **p ≤ 0.01, ***p ≤ 0.001, and ****p ≤ 0.0001 (same for #). For ‘*’ analyses, one-way ANOVA was used for normally distributed data, while Kruskal-Wallis test was used for non-parametric distributions; for ‘#’ analyses, unpaired t-test was used for normally distributed data, while Mann-Whitney test was used for non-parametric distributions.

To evaluate *D. melanogaster*’s innate immune response to infection according to treatment, patterns of gene expression of male and female flies were analyzed at 24-hours post-infection. Results for the 48-hour regimens are shown in **Figure 6**, while results for the 24-hour regimens in **Supplementary Figure S6**.

Interestingly, the inhibition of the pathogen growth caused by the hk treatment triggered the same AMP production that the control group in which *C. albicans* 4372 experiments an extraordinary growth. This response also presents sexual dimorphism. On one hand, male response is Toll-based, further reinforcing the trend prompted by hk priming. On the other, females appear to prioritize the Imd response. Regarding *upd3*, there is not such a difference, and in both cases, it appears to be enhanced in the group primed with hkCa 4372. In contrast, infection with *C. albicans* ATCC has no impact in the innate response. No significant differences were detected in *duox* expression levels (data not shown).

**Figure 7:**
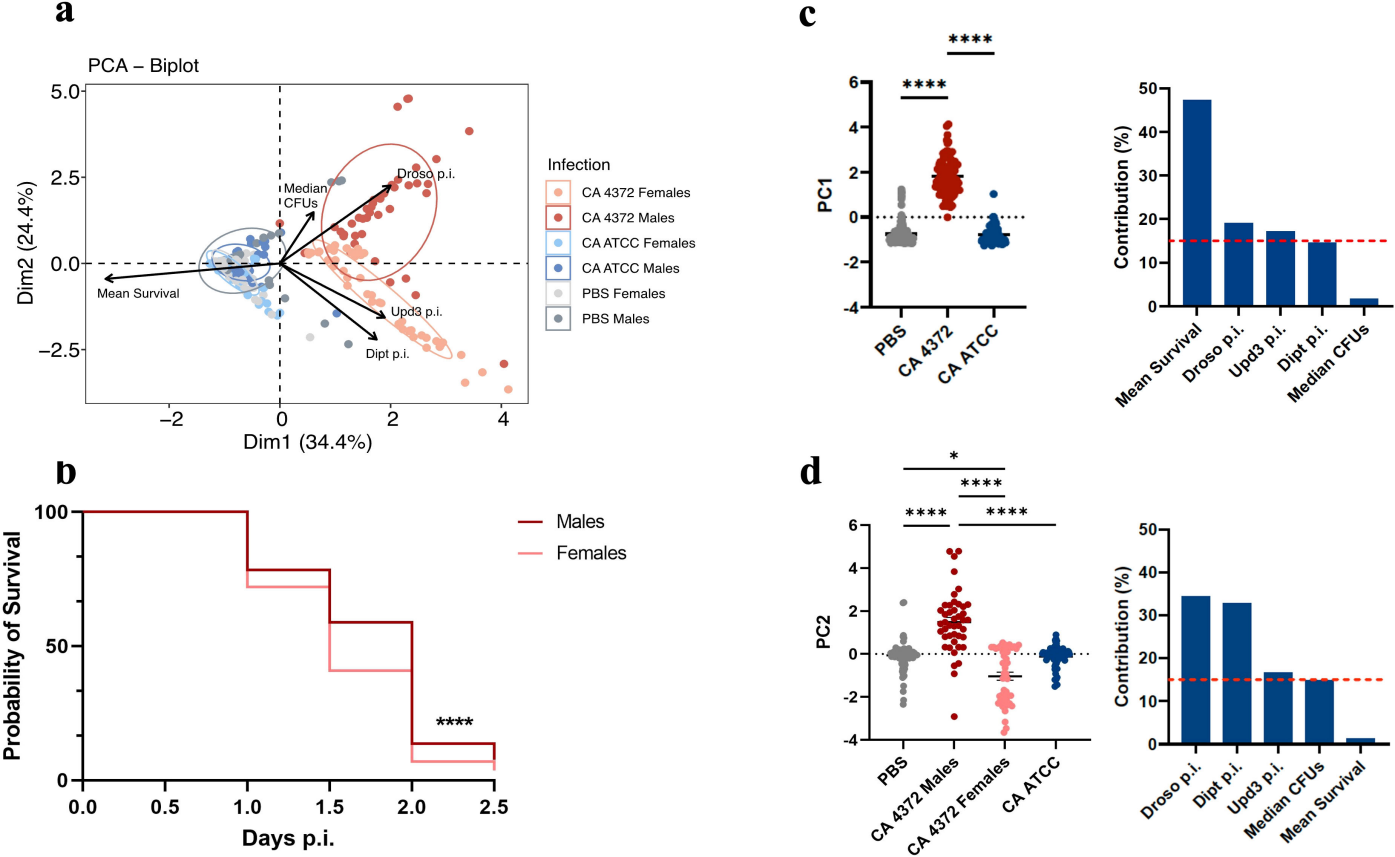
Heterogeneity of antimicrobial peptide gene expression in response to different *C. albicans* infections. **(A)** Principal component analysis (PCA) based on mean survival, median CFUs, and antimicrobial peptide gene expression in male and female flies infected with *C. albicans*. Infection by *C. albicans* ATCC presents similar characteristics to the negative control, while infection by *C. albicans* 4372 is characterized by high AMP induction. **(B)** Sex-related survival diversity in response to infection with *C. albicans* 4372. Female flies have a shorter lifespan compared to males (****p ≤ 0.0001; Log-rank -Mantel-Cox- test). Since this phenomenon is consistent across all treatment regimens, data from each regimen were combined to generate this graph. **(C)** PC1 scores (left) and variable contributions of Dim1 (right). **(D)** PC2 scores (left) and variable contributions of Dim2 (right). Each circle represents an individual fly, and lines depict the means in each group. Statistically significant differences were represented as follows: *p ≤ 0.05 and ****p ≤ 0.0001 (one-way ANOVA for normally distributed data; Kruskal-Wallis test for non-parametric distributions).

Moreover, PCA analysis (**Figures 7A, C, D**) shows a clear pattern, in which survival is the main factor that clusters the control and *C. albicans* ATCC-infected groups in one corner of the graph, while flies infected with *C. albicans* 4372 are placed in the opposed corner of the graph. Dimension 2, on the other hand, emphasizes the sexual dimorphism upon *C. albicans* 4372 infection, marked by *drosomycin* expression in males, and *diptericin* and *upd3* in females. This difference might account for the slightly better survival rate in males (**Figure 7B**). Since no significant differences were observed between the 24- and 48-hour treatment regimens of each treatment group (**Supplementary Figure S3**), results were pooled to simplify data visualization.

## 4 Discussion

The results documented here advance our understanding regarding host-pathogen interactions in *D. melanogaster* and the innate immune mechanisms triggered in response to infection after priming flies with heat-killed *M. manresensis* and *C. albicans*. First, administration of hkMm induces *nox-*dependent production of ROS 72-hours after the end of the treatment, while hkCa prompts repression of *duox* expression. Second, hkCa ATCC and hkCa 4372 treatments induce similar gene expression patterns, while hkMm presents a distinct induction profile. Third, administration of the tested treatments does not result in significant differences in survival among groups. Instead, fly lifespan is highly dependent on the infecting microorganism, with *C. albicans* 4372 presenting a highly virulent profile. Fourth, treatment with hkMm or hkCa leads to CFU clearance in both *C. albicans* ATCC and *C. albicans* 4372 infection groups, even though survival remains unchanged. Fifth, infection with *C. albicans* 4372 induces sexually-dimorphic and extensive *diptericin, drosomycin*, and *upd3* expression in both males and females, while *C. albicans* ATCC does not prompt a significative increase in AMP gene expression.

Our characterization of the effect that the oral treatments have on *D. melanogaster* reveals a delayed activation of the innate immune response in treated flies compared to those that do not receive treatment. This response could be categorized as priming based on the classification established by Divangahi et al. (12), since the immune response remains activated before the second challenge (i.e., infection with *C. albicans*). Previous studies showed that the activation, duration, and specificity of the response triggered are dependent on the microbe used for said priming (1, 2, 9, 25, 26). However, in this case we prove that oral administration of hkMm provides unspecific (i.e., heterologous) protection against infection by *C. albicans*.

Our data also shows that hkMm triggers *nox*-dependent production of ROS at 72 hours after the end of the treatment, while no *duox* induction was detected in any of the tested regimens. In fact, *duox* expression was repressed in the hkCa treatments in both sexes and analyzed time-points. Typically, expression of *nox* has been related to a response to commensal bacteria and tissue homeostasis, while *duox* has been linked to invading pathogens and activation of the immune response (27, 28). Thus, the aforementioned *nox* induction upon hkMm administration together with the lack of *duox* activation suggest that all tested oral treatments induce a homeostatic response in the gut of *D. melanogaster*. This hypothesis is supported by the additional induction of *upd3*, which has been previously linked to intestinal epithelia renewal and homeostasis (29–32).

We also show that host sex is a key factor for the priming outcome, and that sexual dimorphism is present in both hkMm and hkCa treatment regimens, a phenomenon similar to that described by Wen et al. (33) when priming flies with heat-killed *Staphylococcus aureus*. These differences could be partly explained by the dimorphic function of the Toll pathway (34, 35). Our results also indicate a 24-hour delay in *diptericin* and *upd3* induction in females. Differences in the hormonal milieu and reproductive strategies could explain this difference, since certain stages of the reproductive cycle could prioritize reproductive functions over immune responses and reproductive output has not been thoroughly examined in a priming context (1, 36).

The aforementioned sexual dimorphism becomes even more evident after infection with a virulent *C. albicans* isolate. The Imd and Toll signaling pathways are the main actors in the activation of humoral antimicrobial defenses in the fat body of *D. melanogaster* (18–20, 37, 38). In line with the hypothesis that Toll is needed to confer the priming-induced protection and to combat *C. albicans* infection (38, 39), this pathway is strongly activated upon *C. albicans* inoculation. Similarly to what was described by Patrnogic et al. (40), we found that pre-exposure to the heat-killed *C. albicans* and *M. manresensis* increases AMP gene expression but fails to confer a survival advantage upon infection with *C. albicans*. These results contradict the findings of Pham et al. (25), in which flies primed with *Streptococcus pneumoniae* display both infection clearance and an increase in lifespan. Moreover, female flies die at a significatively faster rate than males, while presenting lower CFU counts. This contradicts the findings of Belmonte et al. (35), in which they described how male flies tend to be more susceptible to *C. albicans* infection. The decrease in CFU counts detected in females could be related to higher *diptericin* induction. However, various studies on *Candida* spp. virulence in *D. melanogaster* suggest that AMP expression is not what primarily prompts infection clearance, but instead phagocytes are the critical effectors of the primed response subsequent to infection (25, 39, 41, 42). We hypothesize that, even if AMPs are not completely accountable for infection clearance, they could be related to the decrease in lifespan. Several studies support this hypothesis, since previous evidence indicates that a high activation of the immune response can be associated with trade-offs that impact fly lifespan (1, 9, 43, 44). Moreover, the aforementioned survival advantage described by Pham et al. for *S. pneumoniae* could be explained by the fact that this microbe is a poor AMP inducer in contrast to the highly-inductive *C. albicans* 4372 (25, 38). Thus, future studies should therefore focus on determining the role of the cellular response in this particular infection, as well as on the role of trade-offs in response to infection and fly lifespan.

Another intriguing finding of this study is the unexpected high virulence of the *C. albicans* 4372 isolate. Previous studies report an average fly lifespan of 2-3 days upon inoculation of 2,000-5,000 CFUs/fly (45, 46), while in this case flies injected with 50 CFUs die within the first two-days post-infection. In addition, we also observe a higher growth capacity of this strain, reaching 5 logs in 24 hours, compared to the control ATCC, which reaches a maximum of 2 logs even after 10 days. We examined various hypotheses aimed at elucidating the underlying mechanisms responsible for this phenomenon. The first hypothesis is based on the production of a certain virulence factor by this specific isolate. *C. albicans* virulence factors are primarily released via extracellular vesicles (EVs), and they play a crucial role in yeast-to-hypha transition and in regulating host immunity (47–49). Thus, the microbe could be inducing systemic hyphal growth and protein secretion upon infection, resulting in extensive fly death. Nevertheless, more research should be conducted to fully characterize the secretome and the proteomic profile of this isolate. Moreover, Silao et al. recently described proline as a preferred energy source for *C. albicans*, and how proline catabolism is required for invasive growth (50, 51). Since the haemolymph of *D. melanogaster* is rich in arginine (52) and arginine metabolism and proline biosynthesis are interconnected due to shared metabolic pathways, the second hypothesis is based on an upregulation in proline utilization genes (PUT) in this isolate that could be related to higher virulent properties (51). However, future research will be focused on the genomic characterization of *C. albicans* 4372 isolate to study the expression levels of its putative virulence genes and determine if they are differentially expressed during the infective process.

Finally, our last hypothesis builds upon the host’s reaction as the main cause of fly death after infection, in particular the induction of a stormy innate immune response. A study by Brandt et al. supports this premise, since they demonstrate how *Salmonella-*infected flies die from metabolic collapse rather than from the direct result of microbial action (53). Nonetheless, the fact that flies did not die upon infection with hkCa 4372 indicates that some factor only present while the pathogen is alive is required for the previously stated substantial activation of the immune system. Accordingly, future studies should focus on deciphering which are these specific factors and how they relate with host’s response to infection.

All in all, the provided data herein suggest that priming with oral administration of a low dose treatment based on heat-killed *M. manresensis* or *C. albicans* could induce increased resistance against subsequent infections of *C. albicans* based only on the innate immune response. This increased resistance has no impact on the host survival. Intriguingly, we have detected a virulence factor linked to a specific virulent strain which requires its living presence to stimulate a stormy innate response similarly to the one induced after its exacerbated growth in non-primed flies. This mechanism will merit future in-depth analysis.

## Data Availability

The raw data supporting the conclusions of this article will be made available by the authors, without undue reservation.
