# Supplementary material for: Candida albicans infection model in Drosophila melanogaster suggests a strain-specific virulent factor boosting a stormy innate immune response
Source: Front Immunol. 2024 Oct 31;15:1474516. doi: 10.3389/fimmu.2024.1474516 (PMC11560421; doi:10.3389/fimmu.2024.1474516)
Supplement: Supplementary file 1 [file DataSheet1.pdf]

Supplementary files

Candida albicans infection model in *Drosophila melanogaster* suggests a strain-specific virulent factor boosting a stormy innate immunity response

Mariona Cortacans[1-4], Marta Arch[1-4], Esther Fuentes[1,2,4], Pere-Joan Cardona[1-5*]

[1]Servei de Microbiologia, LCMN, Hospital Universitari Germans Trias i Pujol (HUGTiP), Badalona (08916), Spain

[2]Experimental Tuberculosis Unit (UTE). Institut de Recerca Germans Trias i Pujol (IGTP), Badalona (08916), Spain

[3]Microbiology and Genetics Department, Universitat Autònoma de Barcelona, Bellaterra (08193), Spain

[4]Centre de Medicina Comparativa i Bioimatge de Catalunya (CMCiB); Badalona (08916), Spain

[5]Centro de Investigación Biomédica en Red de Enfermedades Respiratorias (CIBERES), Madrid (28029), Spain

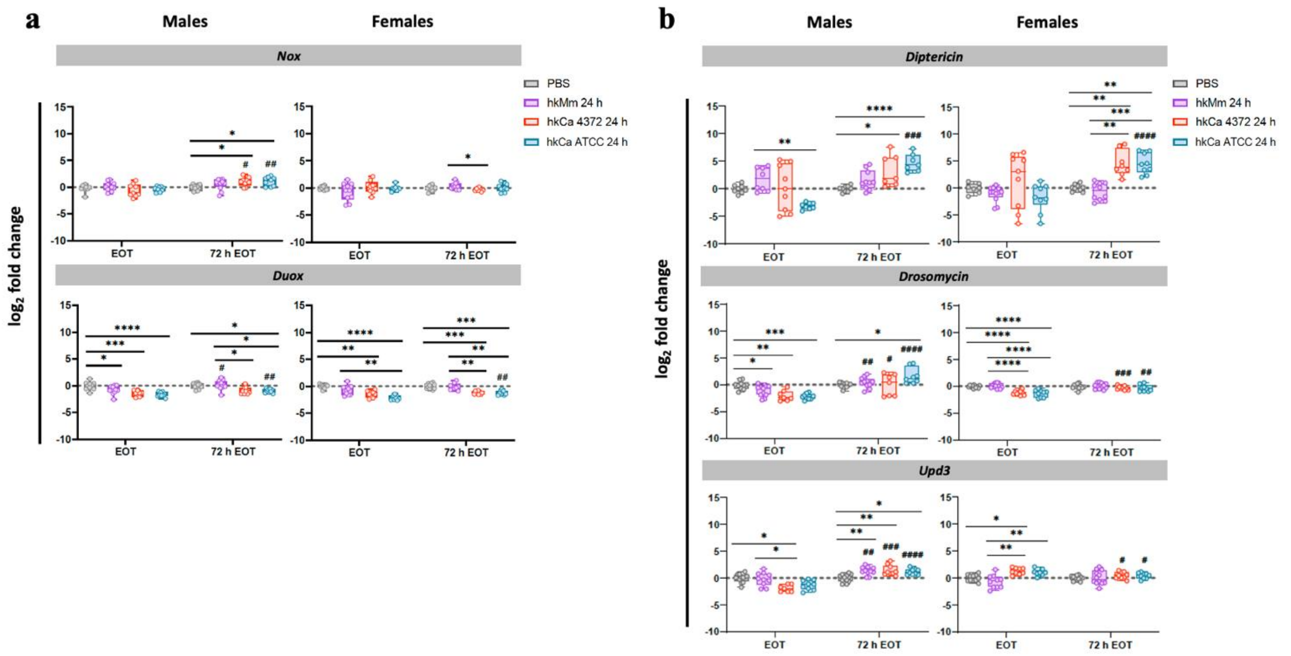

Supplementary Figure 1: Gene expression analysis in response to the 24-hour treatment regimens. (a) Nox and Duox. (b) *Diptericin*, *drosomycin*, and *upd3*. Real-Time qPCR results were normalized with the *rpl32* gene and presented as the $\log_2$ fold change between the PBS-injected and pathogen-infected groups. Each dot represents the relative expression of the corresponding gene from a pool of three flies. The dashed line in each graph represents the controls' relative expression with a fold change of 1. '*' indicate differences between treatments within each time-point, while '#' represent differences between each 72 h EOT sample and their respective EOT time-point. Data was analysed for normality and significant differences were represented as follows: *$p \leq 0.05$, **$p \leq 0.01$, ***$p \leq 0.001$, and ****$p \leq 0.0001$ (same for #). For '*' analyses, one-way ANOVA was used for normally distributed data, while Kruskal-Wallis test was used for non-parametric distributions; for '#' analyses, unpaired t-test was used for normally distributed data, while Mann-Whitney test was used for used for non-parametric distributions.

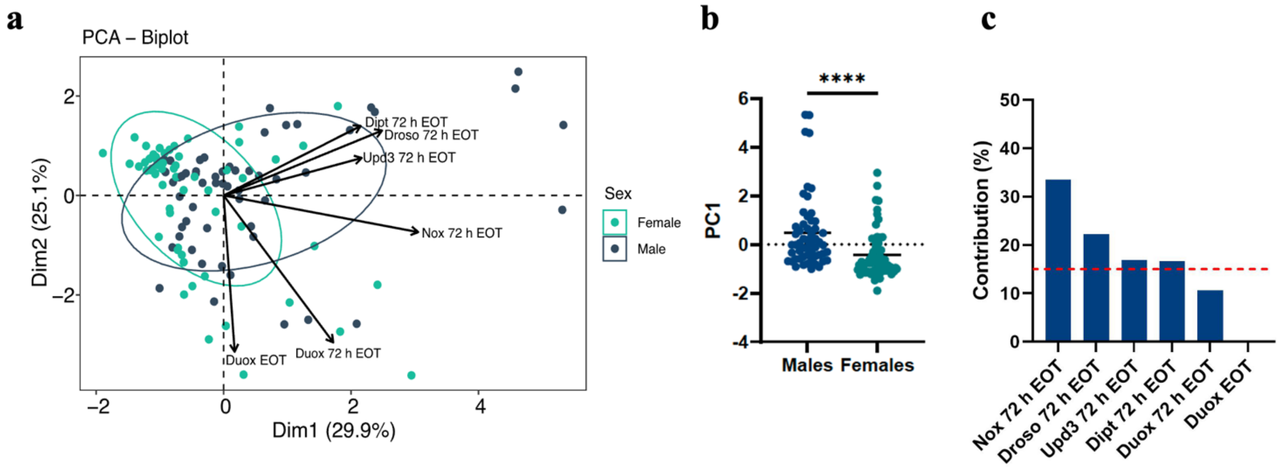

Supplementary Figure 2: Heterogeneity of antimicrobial peptide gene expression between male and female flies in response to oral treatment. (a) Principal component analysis (PCA) based on expression of selected genes in male and female flies treated with hkMm and hkCa. (b) PC1 scores. Each circle represents an individual fly, and lines depict the means in each group. Male and female flies present statistically significant differences regarding response to treatment data distribution in Dim1 (****$p \leq 0.0001$; Mann-Whitney test). (c) PC1 variable contributions.

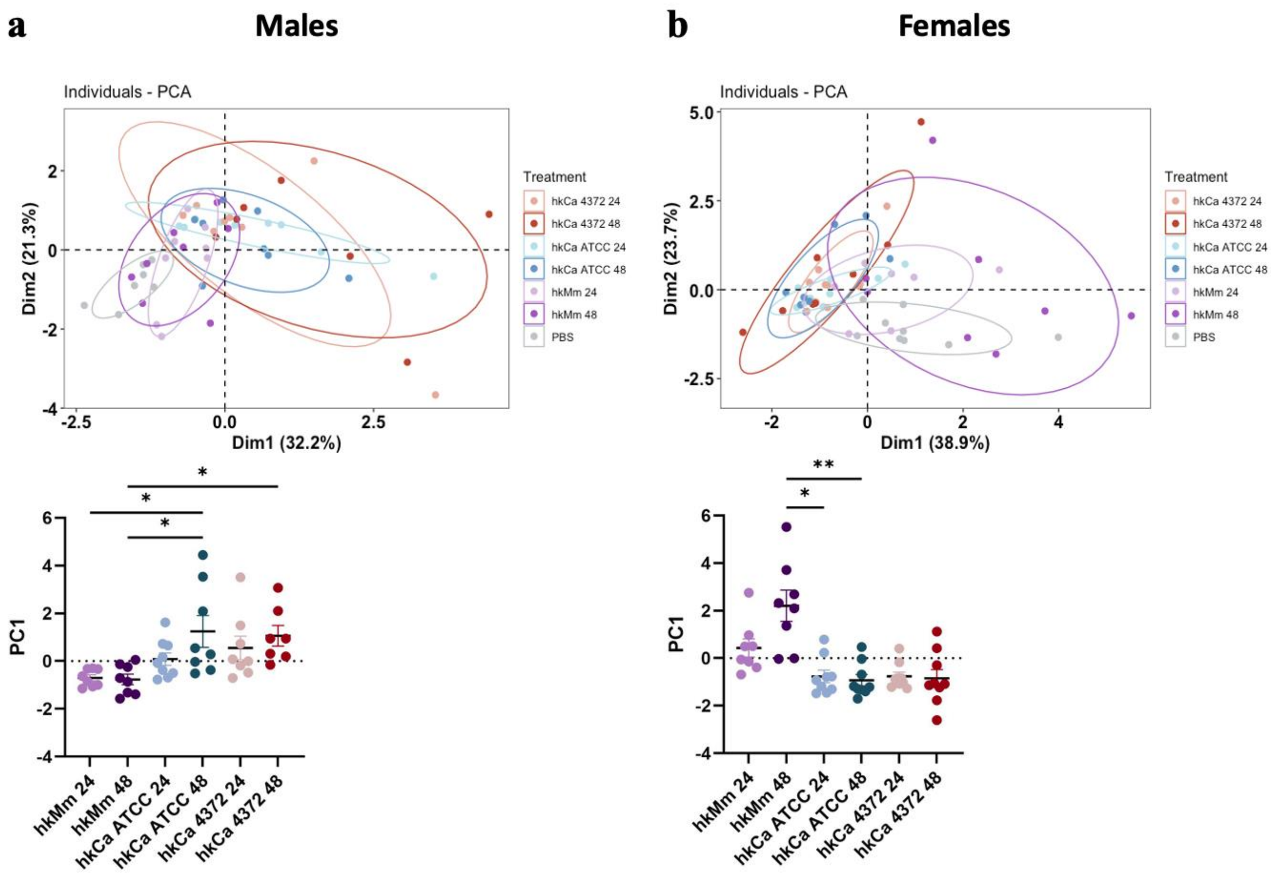

Supplementary Figure 3: Differences between the 24- and 48-hour regimens of each treatment group. Principal component analysis (PCA) (Top) and PC1 scores (Bottom) of males (a) and females (b) based on expression of selected genes in flies treated with hkMm and hkCa according to the duration of the treatment. The overall tendency in both sexes is the absence of significant differences between the 24- and 48-hour regimens. Each circle represents an individual fly, and lines depict the means in each group. Data was analysed for normality and significant differences were represented as follows: $*p \leq 0.05$, $**p \leq 0.01$ (one-way ANOVA for normally distributed data and Kruskal-Wallis for non-parametric distributions).

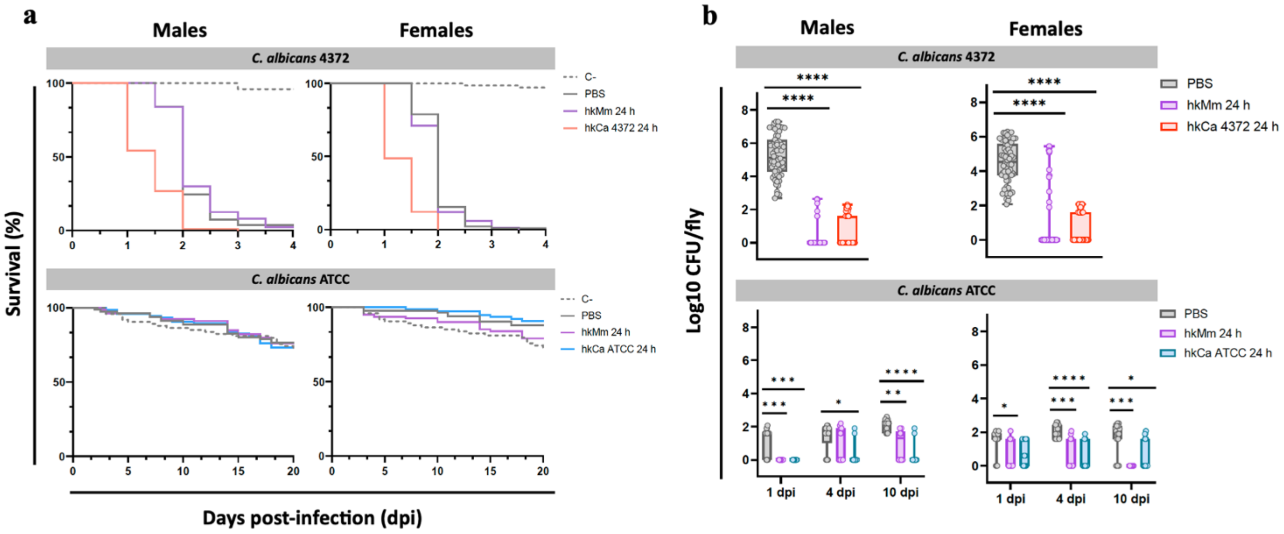

Supplementary Figure 4: Rapid fly death due to infection with *C. albicans* 4372 and infection clearance in response to treatment. (a) Survival curves of flies infected with *C. albicans* 4372 and *C. albicans* ATCC (24-hour treatment regimens). *C. albicans* 4372 (Top) presented a highly virulent profile in both males (left) and females (right), while *C. albicans* ATCC (Bottom) displayed an avirulent phenotype. (b) Pathogen load of flies infected with *C. albicans* 4372 and *C. albicans* ATCC (24-hour treatment regimens). Pathogen load is expressed as Log_{10} CFUs/fly and each circle represents an individual fly. The limit of detection for bacterial colonies in each sample is set at 40 CFUs. Absence of CFUs was computed as a 1 instead of 0 to improve data visualisation. (Top) Within the *C. albicans* 4372 infection group, pathogen load is represented as the Log_{10} CFUs counts at the moment of fly death. (Bottom) Within the *C. albicans* ATCC infection group, multiple time-points were set to determine pathogen load of live flies. Statistically significant differences are represented as follows: $*p \leq 0.05$, $**p \leq 0.01$, $***p \leq 0.001$, and $****p \leq 0.0001$ (one-way ANOVA for normally distributed data; Kruskal-Wallis test for non-parametric distributions).

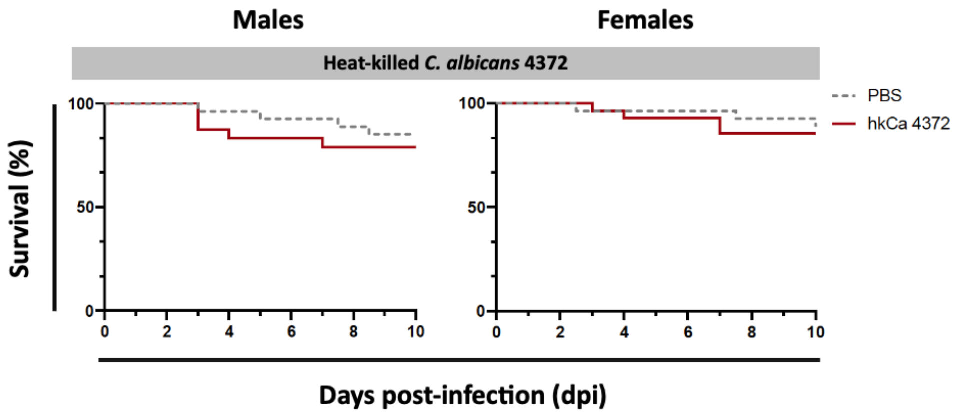

Supplementary Figure 5: Survival curves of flies infected with hkCa 4372 (preliminary analysis). Male and female flies infected with hkCa 4372 display survival curves that are mostly equivalent to those of the control. These results correspond to a single replicate consisting of 30 males and 30 females.

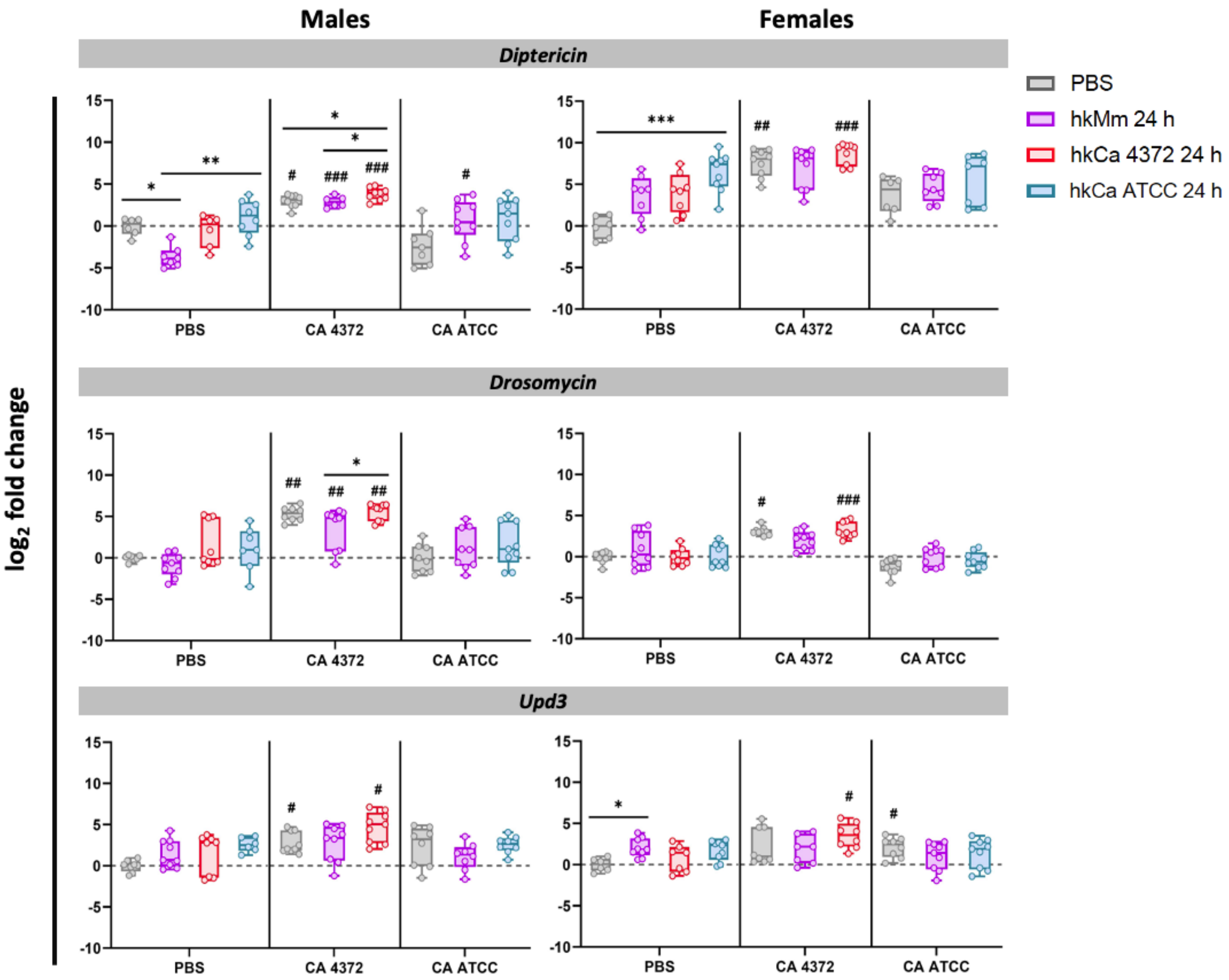

Supplementary Figure 6: *Diptericin*, *drosomycin*, and *upd3* induction in response infection (24-hour treatment regimens). *C. albicans* 4372 prompted high *diptericin*, *drosomycin*, and *upd3* expression in both males and females, in contrast to *C. albicans* ATCC. Expression levels were calculated using the $2^{-\Delta\Delta CT}$ method with *rpl32* gene for normalization. '*' indicate differences between treatments within each time-point, while '#' represent differences with their relative uninfected control after the treatment to discern expression variations due to infection. Each dot represents the relative expression of the corresponding gene from a pool of three flies. The dashed line in each graph represents the controls' relative expression with a fold change of 1. Data was analysed for normality and statistically significant differences are represented as follows: $^{*}p \leq 0.05$, $^{**}p \leq 0.01$, $^{***}p \leq 0.001$, and $^{****}p \leq 0.0001$ (same for #). For '*' analyses, one-way ANOVA was used for normally distributed data, while Kruskal-Wallis test was used for non-parametric distributions; for '#' analyses, unpaired t-test was used for normally distributed data, while Mann-Whitney test was used for used for non-parametric distributions.
